# Evaluation of the safety and efficacy of using human menstrual blood‐derived mesenchymal stromal cells in treating severe and critically ill COVID‐19 patients: An exploratory clinical trial

**DOI:** 10.1002/ctm2.297

**Published:** 2021-01-27

**Authors:** Xiaowei Xu, Wanli Jiang, Lijun Chen, Zhenyu Xu, Qiang Zhang, Mengfei Zhu, Peng Ye, Hang Li, Liang Yu, Xiaoyang Zhou, Chenliang Zhou, Xiaobei Chen, Xiaoqin Zheng, Kaijin Xu, Hongliu Cai, Shufa Zheng, Wubian Jiang, Xiaojun Wu, Dong Li, Lu Chen, Qingqing Luo, Yingyan Wang, Jingjing Qu, Yifei Li, Wendi Zheng, Yingan Jiang, Lingling Tang, Charlie Xiang, Lanjuan Li

**Affiliations:** ^1^ State Key Laboratory for Diagnosis and Treatment of Infectious Diseases National Clinical Research Center for Infectious Diseases Collaborative Innovation Center for Diagnosis and Treatment of Infectious Diseases The First Affiliated Hospital College of Medicine Zhejiang University Hangzhou Zhejiang P. R. China; ^2^ Department of Infectious Diseases Renmin Hospital of Wuhan University Wuhan Hebei P.R. China; ^3^ Innovative Precision Medicine (IPM) Group Hangzhou Zhejiang P. R. China; ^4^ Shulan (Hangzhou) Hospital, Zhejiang Shuren University Shulan International Medical College Hangzhou Zhejiang P. R. China; ^5^ Department of Respiratory Disease Thoracic Disease Centre The First Affiliated Hospital College of Medicine Zhejiang University Hangzhou Zhejiang P. R. China

**Keywords:** coronavirus disease 2019 (COVID‐19), mesenchymal stromal cells, safety and efficacy, severe and critical patients

## Abstract

The coronavirus disease 2019 (COVID‐19), caused by severe acute respiratory syndrome coronavirus 2 (SARS‐CoV‐2) was identified in December 2019 and has subsequently spread worldwide. Currently, there is no effective method to cure COVID‐19. Mesenchymal stromal cells (MSCs) may be able to effectively treat COVID‐19, especially for severe and critical patients. Menstrual blood‐derived MSCs have recently received much attention due to their superior proliferation ability and their lack of ethical problems. Forty‐four patients were enrolled from January to April 2020 in a multicenter, open‐label, nonrandomized, parallel‐controlled exploratory trial. Twenty‐six patients received allogeneic, menstrual blood‐derived MSC therapy, and concomitant medications (experimental group), and 18 patients received only concomitant medications (control group). The experimental group was treated with three infusions totaling 9 × 10^7^ MSCs, one infusion every other day. Primary and secondary endpoints related to safety and efficacy were assessed at various time points during the 1‐month period following MSC infusion. Safety was measured using the frequency of treatment‐related adverse events (AEs). Patients in the MSC group showed significantly lower mortality (7.69% died in the experimental group vs 33.33% in the control group; *P* = .048). There was a significant improvement in dyspnea while undergoing MSC infusion on days 1, 3, and 5. Additionally, SpO_2_ was significantly improved following MSC infusion, and chest imaging results were improved in the experimental group in the first month after MSC infusion. The incidence of most AEs did not differ between the groups. MSC‐based therapy may serve as a promising alternative method for treating severe and critical COVID‐19.

## Graphical abstract

This prospective and systematic report assessed the ability of menstrual blood‐derived MSCs to treat both severe and critically ill COVID‐19 patients.

MSC‐based therapy may serve in future clinical applications as an alternative way for the treatment of COVID‐19

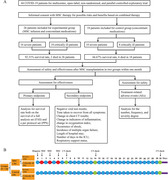

## 1 INTRODUCTION

The coronavirus disease 2019 (COVID‐19), caused by severe acute respiratory syndrome coronavirus 2 (SARS‐CoV‐2), was initially identified in December 2019 as causing a cluster of respiratory infections.^1^, ^2^ COVID‐19 quickly attracted global concern and panic since it is highly contagious.^3^, ^4^, ^5^ Due to a lack of adequate awareness in the first few weeks of the outbreak, the number of infected patients increased swiftly, rapidly spreading to more and more countries.^6^, ^7^ As of January 7, 2021, there have been over 85 929 000 confirmed cases of COVID‐19 worldwide, leading to 1 876 100 deaths (https://www.who.int/emergencies/diseases/novel-coronavirus-2019). Currently, the number of infected patients is still increasing worldwide.

COVID‐19 has an incubation period which can range from 1 to 14 days but usually ranges from 3 to 7 days.^8^ The main symptoms are fever, headache, dry cough, and chest tightness.^9^, ^10^, ^11^ Many patients also experience a sore throat, diarrhea, nasal congestion, and rhinorrhea.^12^, ^13^ Severe patients often develop expiratory hyperextension and dyspnea 1 week after the onset of the disease. In the most severe cases, patients can quickly develop acute respiratory distress syndrome (ARDS), severe acute lung injury, septic shock, metabolic acidosis, and coagulopathy, as reported in a biopsy and autopsy study.^14^ COVID‐19 can easily cause expiratory dyspnea and ARDS. Of the COVID‐19 patients, 13.8% of the cases were severe, 6.1% cases were critical, and about 2.3% cases had fatal outcomes.^15^, ^16^ Since no effective or authorized vaccines are available for preventing COVID‐19 infections, a breakthrough in the therapeutic strategy is vital for the treatment of COVID‐19 and especially for severe or critically ill patients who may develop ARDS and/or expiratory dyspnea.^17^, ^18^, ^19^, ^20^, ^21^ Currently, a few drugs (such as remdesivir and dexamethasone) have shown positive preliminary results in randomized, controlled, open‐label, clinical trials.^22^, ^23^ Even more excitingly, COVID‐19 vaccines with acceptable safety, tolerability, and immunogenicity have been reported by Zhu et al^24^ and Folegatti et al^25^ as being effective in initial human clinical trials. Apart from these, many other groups are also developing available vaccines such as the BNT162 mRNA vaccine^26^ sponsored by Pfizer Inc. and BioNTech SE; the chimpanzee adenovirus vectored vaccine ChAdOx1 nCoV‐19 (AZD1222)^27^, ^28^ sponsored by AstraZeneca; the mRNA‐1273 vaccine^29^ codeveloped by the Cambridge, Massachusetts‐based biotechnology company Moderna, Inc., and the National Institute of Allergy and Infectious Diseases (NIAID); the adenovirus serotype 26 (Ad26) vaccine^30^ developed by Janssen Vaccines & Prevention B.V.; the BBIBP‐CorV vaccine^31^ developed by the Beijing Institute of Biological Products; the CoronaVac vaccine^32^ developed by Sinovac Life Sciences; and the NVX‐CoV2373 vaccine^33^ sponsored by Novavax, Inc. However, no specific drugs have been reported to be absolutely effective in treating COVID‐19. Moreover, SARS‐CoV‐2‐induced secondary infections have been reported to induce multiple organ dysfunction syndrome in severe or critically ill patients, and this issue remains a serious problem worldwide.

Mesenchymal stem cells have been used for almost three decades, and have made great progress.^34^ According to the recommendations of the International Society for Cell & Gene Therapy (ISCT) in 2019, mesenchymal stem cells should be named mesenchymal stromal cells (MSCs).^35^ MSC‐based cellular therapy has been the subject of an increasing number of studies due to the cells’ self‐renewing capacity, multipotent potential, low immunogenicity, anti‐inflammatory activity, and ability to home to damaged tissue.^36^, ^37^, ^38^ More importantly, MSCs have unique immunomodulatory functions of both innate and adaptive immune responses, making them an attractive cell therapy tool.^38^, ^39^ MSCs regulate adaptive immune responses mainly through targeting T lymphocytes, B lymphocytes, antigen‐presenting cells (APCs), dendritic cells (DCs), natural killer (NK) cells, and regulatory T cells (Tregs).^38^ MSCs also regulate innate immune responses mainly through targeting DCs, NK cells, innate TH17 cells, neutrophils, monocytes, macrophages, and mast cells.^39^ Further, MSC‐based therapies have shown promising results in several clinical studies across a variety of diseases.^40^, ^41^ With the development of stem cell research, researchers have found that after injecting MSCs, the human body activates the host's innate immune cascade system, such as complement and blood coagulation, which is defined as the instant blood‐mediated inflammatory response (IBMIR).^42^ IBMIR is of key importance considering the highly procoagulant state of many critically and severely ill patients in need of MSC therapy.^43^ MSCs can be obtained from various sources, including bone marrow (BM), adipose (AD), umbilical cord (UC), placenta, menstrual blood, muscle, dental pulp, Wharton's jelly (WJ), fetal liver, amniotic membrane, amniotic fluid, urine etc.^44^, ^45^, ^46^, ^47^, ^48^ Furthermore, MSC‐based treatment has demonstrated promising results in studies on inflammatory lung disease, showing an ability to inhibit alveolar collapse, cell apoptosis, and collagen accumulation in lung tissues.^49^ The angiotensin‐converting enzyme 2 (ACE2) is identified as a receptor of SARS‐CoV‐2 entering into target cells.^50^, ^51^ In addition, researchers have demonstrated that MSCs do not express ACE2, and MSCs are resistant to SARS‐CoV‐2 infection as well as when exposed to SARS‐CoV‐2‐infected cells.^52^, ^53^ Additionally, following infusion of allogeneic MSCs into nine patients with ARDS, Wilson et al^54^ observed no prespecified side effects including cardiac arrhythmia, hypoxemia, and ventricular tachycardia. Further, our team reported that MSC transplantation significantly lowers the mortality of epidemic Influenza A (H7N9)‐induced ARDS patients.^55^ BM‐MSCs have been shown to improve repair after ventilator‐induced lung injury, to facilitate the resolution of inflammation, and to restore lung function and structure in ARDS patients.^56^ With regard to the COVID‐19 epidemic, MSCs from different sources (especially UC‐MSCs) have been used in clinical trials.^53^, ^57^ A good proliferation rate plays an important role in clinical application, because stem cell‐based treatment is dose‐dependent, and usually human clinical research requires millions of stem cells. The doubling time for menstrual blood‐derived MSCs is about 20 h, and the doubling time for BM‐MSCs is about 40‐45 h. Thus, MSCs from menstrual blood can obtain a better yield within a shorter time at early passages.^58^, ^59^ More importantly, menstrual blood‐derived MSCs offer an alternative that is both painless and free of the ethical issues that may arise from BM‐MSCs donations.^60^ Thus, menstrual blood‐derived MSC‐based therapy may be a promising treatment for COVID‐19, particularly to combat the inflammatory cytokine storms observed in severe and critical patients.

This study is an exploratory trial to assess the ability of menstrual blood‐derived MSCs to treat severe and critically ill COVID‐19 patients. To this end, we assessed the safety, therapeutic efficacy, and tolerability of transplanted MSCs with a 1 month follow‐up after SARS‐CoV‐2 infection. In particular, we assessed any improvements in pulmonary function. Our results not only shed light on the ability of MSCs to treat COVID‐19 patients, but also suggest that MSCs are a promising tool to treat acute or chronic pneumonia in future clinical applications.

## 2 MATERIALS AND METHODS

### 2.1 Study design and participants

This was a multicenter, open‐label, nonrandomized, and parallel controlled phase I clinical trial performed at two major academic centers in China: the Renmin Hospital of Wuhan University, Wuhan and the First Affiliated Hospital, College of Medicine Zhejiang University, Hangzhou. The Shulan (Hangzhou) Hospital, affiliated to Zhejiang Shuren University, Shulan International Medical College, Hangzhou also participated in related studies. Eligible patients were 18‐75 years old and confirmed to be positive for the SARS‐CoV‐2 RNA virus by polymerase chain reaction (PCR) analysis performed within biological safety protection level 3 laboratories at Wuhan University and Zhejiang University. Before the initiation of this study, the research protocol, case report form (eCRF), and informed consent form were each obtained and approved by the Ethics Committees of Renmin Hospital of Wuhan University (WDRY2020‐K011) and the First Affiliated Hospital, College of Medicine, Zhejiang University in accordance with the Declaration of Helsinki and the criteria of Good Clinical Practice.^61^ This clinical trial was also registered in the Chinese Clinical Trial Registry (ChiCTR2000029606). The investigators fully educated each patient's legal representative regarding the informed consent form, the detailed therapeutic procedure, as well as the possible risks and benefits. The patients had the right to withdraw from this clinical study at any time during the clinical trial. Considering the urgency of the COVID‐19 epidemic and the operability of the enrollment process, a randomized table was not used for randomization in the research process at Renmin Hospital of Wuhan University, and cases (including severe and critically ill patients) were matched based on similar severity levels and similar timing of enrollment in the study. This clinical trial was an open study and did not involve blinding or emergency unblinding.

All patients met the diagnostic criteria for COVID‐19 according to the National Health Commission of China (Trial Version 5). Following established clinical guidelines for the diagnosis and treatment of COVID‐19, patients can be classified as mild, common, severe, or critical.^53^, ^62^, ^63^ Only severe and critically ill COVID‐19 patients were included in the present study. Severe patients were defined as those with respiratory distress, respiratory rate ≥30 breaths/min; resting oxygen saturation ≤93%; or arterial blood partial pressure of oxygen (PaO_2_)/fraction of inspiration O_2_ (FiO_2_) ≤300 mmHg (1 mmHg = 0.133 kPa). Critical COVID‐19 patients were defined as those with respiratory failure who required mechanical ventilation, those who had experienced shock, or those for whom a combination of organ failures necessitated monitoring and treatment in the intensive care unit (ICU). Exclusion criteria for this trial were as follows: (1) severe liver disease; (2) long‐term hemodialysis and severe renal impairment or continuous renal replacement therapy; (3) comorbidities that might affect the ability of researchers to determine drug efficacy (mainly malignant tumors, active tuberculosis, interstitial pneumonia, and pulmonary heart disease); (4) treatment with glucocorticoid medications or other immunosuppressive drugs for longer than 2 weeks; (5) history of major surgery within 30 days of screening or presence of an unhealed surgical wound; (6) allergy to any active/inactive ingredients in the study drug; (7) pregnant or breastfeeding; (8) previous history of prothrombotic events (venous thromboembolism/stroke); (9) other circumstances judged by an investigator to preclude participation. These “other circumstances” leading to exclusion from the study included serious AEs for which the investigator judged that the risk of continuing to participate in the trial was too great, as well as the use of other treatments, without authorization and against medical advice, that could have affected the evaluation. These exclusion criteria followed the National Health Commission of China (Trial Version 5). If a patient met all the inclusion or exclusion criteria, he or she was then enrolled in the experimental group (MSC infusion + concomitant medications) or the control group (concomitant medications).

### 2.2 Stratification of disease treatment and concomitant medications

Patients were enrolled and admitted between January and April 2020 at either Renmin Hospital of Wuhan University or the First Affiliated Hospital, College of Medicine, Zhejiang University. A clinical plan was designed for each patient based on their clinical needs. Since the patients in this clinical study were either severe or critical, concomitant medications were allowed, and the details of all additional treatments were recorded. Drugs that were required to treat other diseases were allowed if an investigator judged that the safety and efficacy of the study medications would not be affected. However, the type and dose of the medications were kept as consistent as possible while prioritizing patient safety. For any concomitant medications or other treatments, detailed medication information was recorded on the original medical record and the eCRF.

Of the 44 patients enrolled in this study, 36 were from Wuhan University People's Hospital, including 20 severe patients and 16 critically ill patients. The remaining eight patients were enrolled from the First Affiliated Hospital, College of Medicine, Zhejiang University including six severe patients and two critically ill patients. Due to the high risk of mortality, patients were given the choice as to which group they were placed in, and 26 patients chose to receive the experimental treatment along with the comprehensive treatment, while 18 patients chose to be included in the control group and receive only the comprehensive treatment. The standard of care was consistent between the two hospitals.

### 2.3 MSC preparation, cell transplantation, and subsequent observation

Allogeneic, menstrual blood‐derived MSCs (no. SC0100919001, no. SC0100919004, and no. SC0100919005 provided by Innovative Precision Medicine (IPM) Group, Hangzhou, China) were obtained from three healthy female donors (age range, 20‐45 years), and the volunteers were educated and provided signed, informed consent before donation, as described in previous studies.^55^, ^64^ The donation protocol was authorized by the Ethics Committee of Zhejiang University. The mononuclear cells within the menstrual blood were collected and purified, and cell viability was measured prior to seeding for cell culture according to the staining with trypan blue solution (Thermo Fisher Scientific, No. 15250061). MSCs were passaged at 70‐80% confluence. Surface‐labeled molecules (including CD29, CD34, CD45, CD73, CD90, CD105, CD117, and HLA‐DR) were measured using flow cytometry (CytoFLEX LX Flow Cytometer, Beckman) and detailed procedures were described in a previous study.^65^ Supporting information Table S1 includes detailed information on the antibodies used for surface marker analysis. Detection of the differentiation potential of MSCs into the osteogenic differentiation medium, chondrogenic differentiation medium, and adipogenic differentiation medium A and B (Cyagen Biosciences) and their detailed information has been reported in a previous study.^66^ PCR analysis was used to check the ACE2 expression level of the MSCs. The detailed procedures for PCR analysis have been described in a previous study.^67^ Briefly, cell samples were homogenized in 1 mL of RNAiso Plus (9108, Takara, Japan) to isolate total RNA according to the manufacturer's instructions. RNA was then reverse‐transcribed into cDNA using a FastQuant RT Kit with gDNase (KR106, Tiangen Biotech, China). Then a total of 10 μL sample (1 μL cDNA, 5 μL PCR Mastermix [KT201, Tiangen Biotech], 1 μL forward primer, 1 μL reversed primer, and 2 μL ddH_2_O) was used for PCR with 30 cycles. Supporting information Table S2 includes the primer sequences for PCR analysis. The resulting cryopreserved MSCs were shipped frozen to the hospitals in a validated liquid nitrogen (≤−135°C) dry shipper. Before their use, MSCs were resuspended in Plasma‐Lyte 148 at room temperature by a local laboratory with a specialized cellular therapy center, and the control group was also administered the same volume of Plasma‐Lyte 148. The viability of MSCs for the three donors should be >90%, which was a criterion for use in the clinical study guided by the Innovative Precision Medicine (IPM) Group. MSCs were used for treatment at the fifth passage, as described in our previous report.^66^

Twenty‐six patients received MSC transplantation, and 18 patients received all treatments except MSC transplantation. Complete case enrollment details and disease severity frequencies of the 44 COVID‐19 patients included in the present study can be viewed in Table 1. Each COVID‐19 patient in the experimental group used the MSC sample from one donor for all three treatment injections. Doctors observed hemodynamic and respiratory parameters at the bedside for at least 1 h to ensure that each patient was stable before MSC transplantation. Then, the MSC infusion was initiated using a standard blood filter tube. A researcher remained at each patient's bedside to continuously monitor the patient for any adverse reactions during the 24 h following treatment. Based on data from studies on the use of MSCs to treat H7N9‐induced ARDS,^55^ MSCs were administered as three infusions totaling 9 × 10^7^ MSCs every other day (day 1, day 3, and day 5). Each infusion contained 3 × 10^7^ cells resuspended in 500 mL saline solution and was performed at a speed of 30‐40 drops/min for about 15 min, followed by a speed of 100‐120 drops/min for 2 h to retain MSC vitality.

**TABLE 1:** Complete case enrollment and analyze the data set of 44 COVID‐19 patients in experimental group and control group

Subject distribution	Experimental group (N = 26)	Control group (N = 18)	Total
Participants, n (%)	26 (59.09)	18 (40.91)	44 (100.00)
Severe, n (%)	16 (61.54)	10 (38.46)	26 (100.00)
Critical, n (%)	10 (55.56)	8 (44.44)	18 (100.00)
Number of treatment, n (%)	26 (59.09)	18 (40.91)	44 (100.00)
Severe, n (%)	16 (61.54)	10 (38.46)	26(100.00)
Critical, n (%)	10 (55.56)	8 (44.44)	18 (100.00)
Number of people who completed the test, n (%)	25 (58.14)	18 (41.86)	43 (100.00)
Number of people who withdrew from the trial, n (%)	1 (100.00)	0 (0.00)	1 (100.00)
Severe, n (%)	0 (000.00)	0 (0.00)	0 (000.00)
Critical, n (%)	1 (100.00)	0 (0.00)	1 (100.00)
Full analysis set, n (%)	26 (59.09)	18 (40.91)	44 (100.00)
In line with the program set, n (%)	24 (57.14)	18 (42.86)	42 (100.00)
Eliminate the number of trials, n (%)	2 (100.00)	0 (0.00)	2 (100.00)
Severe, n (%)	0 (000.00)	0 (0.00)	0 (000.00)
Critically ill, n (%)	2 (100.00)	0 (0.00)	2 (100.00)
Security Analysis Set, n (%)	26 (59.09)	18 (40.91)	44 (100.00)

*Note*. “n” represents number.

### 2.4 Biological measurements and clinical evaluation indices

Laboratory measurements of blood test results, liver function markers, and inflammatory indicators were carried out at the Medical Inspection Center of the First Affiliated Hospital, College of Medicine, Zhejiang University and Renmin Hospital of Wuhan University. Factors which were investigated for having an association with therapeutic features or outcomes were as following: (1) baseline characteristics including age, underlying conditions, and clinical symptoms; (2) laboratory data and chest computed tomography (CT) data; and (3) concomitant medications for basic therapy, symptomatic treatment, antiviral treatment, antibacterial treatment, hormone therapy, intestinal microbial state regulators, extracorporeal blood purification technology, traditional Chinese medicine treatment (including Jinhua Qinggan granules, Lianhua Qingwen capsules [granules], and Shufeng Detoxification capsules [granules]), etc.

**FIGURE 1:**
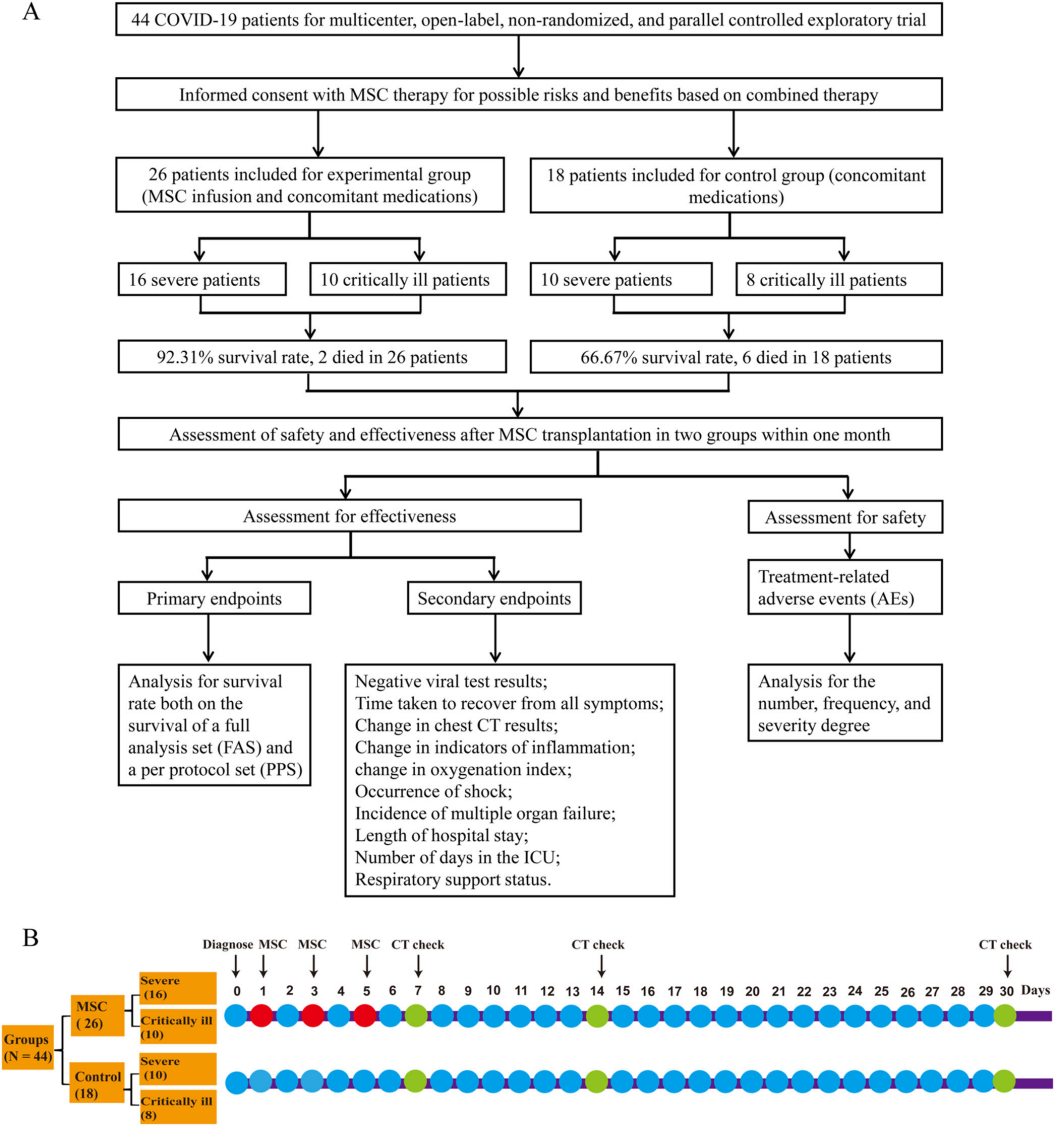
The CONSORT diagram (A) and detail infusions within 1 month (B) for this clinical study. (A) The CONSORT diagram for the clinical trial of 44 severe or critically ill COVID‐19 patients. A sum of 26 patients included for allogeneic, menstrual blood‐derived MSC therapy, and combination therapy (experimental group), among these 16 severe and 10 critically ill patients. Also, 18 patients included for combination therapy (control group), among these 10 severe and 8 critically ill patients. MSC transplantation significantly lower the mortality compared with in control group. Main endpoint and secondary endpoint indexes after MSC transplantation between two groups. And clinical manifestation and chest CT scan for 1 month's follow‐up after MSC transplantation; (B) the detail infusions within 1 month for this clinical study

The objective of the current study was to evaluate the safety and efficacy of MSC transplantation as a treatment for COVID‐19. The primary endpoint of the analysis was survival rate from January to April 2020, and this was based both on the survival of a full analysis set (FAS) and a per protocol set (PPS). The FAS, which was also the effectiveness analysis set, consisted of all 44 subjects who were initially enrolled in this study, which included 26 patients in the experimental group and 18 patients in the control group. Since two cases were initially included in the study who violated the exclusion criteria, the remaining 42 subjects were included in the PPS, which included 24 patients in the experimental group and 18 patients in the control group. The secondary endpoints for this study included measures of effectiveness and tolerability, primarily including negative viral test results, time taken to recover from all symptoms, change in chest CT results, change in indicators of inflammation, change in oxygenation index, occurrence of shock, incidence of multiple organ failure, length of hospital stay, number of days in the ICU, and respiratory support status. Before and after the MSC infusion, patients were tested using several laboratory indices, including those related to hematuria routine, liver and kidney function, coagulation function, vital signs, physical examination, oxygenation (FiO_2_, peripheral oxygen saturation [SpO_2_], oxygen saturation [SaO_2_], and PaO_2_), and inflammatory factors (including interleukin [IL]‐6 and C‐reactive protein [CRP]). Figure 1 shows the CONSORT diagram (Figure 1A) and detailed infusions within 1 month (Figure 1B) for this clinical study. Safety was measured by the frequency of treatment‐related adverse events (AEs) and through careful surveillance of laboratory indices. Clinical data were obtained on each day of MSC infusion (days 1, 3, and 5) as well as on days 7, 14, and 30 of the posttreatment period.

### 2.5 Statistical analysis

To compare the experimental and control groups, *χ*² or Fisher's exact tests were used, as appropriate, for both the FAS analysis and the PPS analysis. The FAS analysis included 44 patients, while the PPS analysis included a subset of 42 patients. The Kaplan–Meier method was utilized to analyze the survival time of discharged patients, and two‐sided 95% exact confidence intervals (CIs) were calculated using the log‐rank test. We also calculated a Cox proportional hazards model to assess factors affecting survival, adjusting for gender, and age as covariates. For the length of hospitalization and ICU stay, a Wilcoxon rank sum test was used to compare the differences between groups. For analysis of inflammatory indices, (including CRP and IL‐6) and oxygenation indices (FiO_2_, SpO_2_, SaO_2_, and PaO_2_), Wilcoxon rank sum tests were used comparing the experimental group before and after the MSC infusion. Statistical analysis was carried out using SAS9.4. *P* value < .05 was considered as statistically significant.

## 3 RESULTS

### 3.1 Patient characteristics and MSC treatment

The viability of MSCs for the three donors was >90% (91% for SC0100919001 and SC0100919004, and 92% for SC0100919005). MSCs strongly expressed CD29, CD73, CD90, and CD105; and MSCs were negative for CD34, CD45, CD117, and HLA‐DR (Supporting information Figure S1). Furthermore, MSCs can be successfully induced into osteogenic, adipogenic, and chondrogenic cells through the specific medium, and the representative picture for each kind of differentiated cells is shown in Supporting information Figure S2. A representative electrophoretogram with two pairs of primers is shown in Supporting information Figure S3. MSC ACE2 expression was negative according to PCR analysis, with ddH_2_O as a negative control and 293T cells as a positive control.

Twenty‐six patients were included in the experimental group and treated with MSC transplantation and combination therapy. Among these, 16 (61.5%) were classified as severe and 10 (38.5%) were classified as critical. Eighteen patients were included in the control group and received only combination therapy. Among these, 10 (55.6%) were classified as severe and 8 (44.4%) were classified as critical (Table 2). The experimental group contained 17 males (65.38%) and nine females (34.62%), while the control group contained 13 males (72.22%) and five females (27.78%). The mean and median age of the experimental group was 58.31 ± 12.49 and 57.50 years, respectively, while the mean and median age of the control group was 61.11 ± 11.03 and 64.00 years, respectively. There were no statistically significant differences with regards to gender or age (*P* > .05). There were no significant differences (*P* > .05) in clinical symptoms (including fever, expiratory dyspnea, sore throat, diarrhea, and chest tightness) between the experimental group and the control group during the baseline period (Table 2). The symptom of cough showed a significant improvement following MSC infusion on day 1 (*P* = .037) compared to that of the control group, but no differences were found at other times points. During the period of MSC infusion (days 1, 3, and 5) and the post‐treatment period (days 7, 14, and 30), the symptoms of fever, cough, sore throat, diarrhea, and chest tightness were not significantly different (Table 2). There was a significant improvement in expiratory dyspnea while undergoing MSC infusion on day 1 (*P* = .016), day 3 (*P* = .040), and day 5 (*P* = .031) compared to the control group (Table 2), but there were no significant differences on day 7 (*P* = .631), day 14 (*P *= .635), or day 30 (*P* = 1.000).

**TABLE 2:** Baseline characteristics of 44 enrolled COVID‐19 patients as well as clinical symptoms at baseline and days 1, 3, 5, 7, 14, and 30 in the experimental group and the control group

Baseline characteristics	Condition, layout	Experimental group (N = 26)	Control group (N = 18)	*P*
Degree of disease	Severe, n (%)	16 (61.50)	10 (55.60)	
	Critically ill, n (%)	10 (38.50)	8 (44.40)	
Age (Years)	Mean ± SD	58.31 ± 12.49	61.11 ± 11.03	.447
	Median (Minimum, Maximum)	57.50 (31.00, 83.00)	64.00 (39.00, 81.00)	
Sex	Male, n (%)	17 (65.38)	13 (72.22)	.632
	Female, n (%)	9 (34.62)	5 (27.78)	
Fever at baseline period	No, n (%)	13 (50.00)	10 (55.56)	.717
	Yes, n (%)	13 (50.00)	8 (44.44)	
Fever at day 1	No, n (%)	21 (84.00)	13 (72.22)	.455#
	Yes, n (%)	4 (16.00)	5 (27.78)	
Fever at days 3	No, n (%)	20 (80.00)	14 (77.78)	1.000#
	Yes, n (%)	5 (20.00)	4 (22.22)	
Fever at days 5	No, n (%)	22 (88.00)	15 (83.33)	.683#
	Yes, n (%)	3 (12.00)	3 (16.67)	
Fever at days 7	No, n (%)	23 (95.83)	12 (80.00)	.279#
	Yes, n (%)	1 (4.17)	3 (20.00)	
Fever at days 14	No, n (%)	20 (95.24)	12 (85.71)	.551#
	Yes, n (%)	1 (4.76)	2 (14.29)	
Fever at days 30	No, n (%)	11 (100.00)	10 (90.91)	1.000#
	Yes, n (%)	0 (0.00)	1 (9.09)	
Cough at baseline period	No, n (%)	10 (38.46)	8 (44.44)	.691
	Yes, n (%)	16 (61.54)	10 (55.56)	
Cough at day 1	No, n (%)	15 (60.00)	5 (27.78)	.037*
	Yes, n (%)	10 (40.00)	13(72.22)	
Cough at days 3	No, n (%)	18 (72.00)	8 (44.44)	.068
	Yes, n (%)	7 (28.00)	10 (55.56)	
Cough at days 5	No, n (%)	16(64.00)	8(44.44)	.203
	Yes, n (%)	9(36.00)	10(55.56)	
Cough at days 7	No, n (%)	20 (83.33)	11(73.33)	.686#
	Yes, n (%)	4 (16.67)	4 (26.67)	
Cough at days 14	No, n (%)	18 (85.71)	11 (78.57)	.664#
	Yes, n (%)	3 (14.29)	3 (21.43)	
Cough at days 30	No, n (%)	9 (81.82)	9 (81.82)	N/A
	Yes, n (%)	2 (18.18)	2 (18.18)	
Expiratory dyspnea at baseline period	No, n (%)	18 (69.23)	10 (55.56)	.354
	Yes, n (%)	8 (30.77)	8 (44.44)	
Expiratory dyspnea at day 1	No, n (%)	20 (80.00)	8 (44.44)	.016*
	Yes, n (%)	5 (20.00)	10 (55.56)	
Expiratory dyspnea at days 3	No, n (%)	21 (84.00)	10 (55.56)	.040*
	Yes, n (%)	4 (16.00)	8 (44.44)	
Expiratory dyspnea at days 5	No, n (%)	22 (88.00)	10 (55.56)	.031#*
	Yes, n (%)	3 (12.00)	8 (44.44)	
Expiratory dyspnea at days 7	No, n (%)	22 (91.67)	13 (86.67)	.631#
	Yes, n (%)	2 (8.33)	2 (13.33)	
Expiratory dyspnea at days 14	No, n (%)	18 (85.71)	13 (92.86)	.635#
	Yes, n (%)	3 (14.29)	1 (7.14)	
Expiratory dyspnea at days 30	No, n (%)	10 (90.91)	11 (100.00)	1.000#
	Yes, n (%)	1 (9.09)	0 (0.00)	
Sore throat at baseline period	No, n (%)	24 (92.31)	16 (88.89)	1.000#
	Yes, n (%)	2 (7.69)	2 (11.11)	
Sore throat at day 1	No, n (%)	25 (100.00)	16 (88.89)	.169#
	Yes, n (%)	0 (0.00)	2 (11.11)	
Sore throat at days 3	No, n (%)	24 (96.00)	18 (100.00)	1.000#
	Yes, n (%)	1 (4.00)	0 (0.00)	
Sore throat at days 5	No, n (%)	24 (96.00)	18 (100.00)	1.000#
	Yes, n (%)	1 (4.00)	0 (0.00)	
Sore throat at days 7	No, n (%)	24 (100.00)	15 (100.00)	N/A
	Yes, n (%)	0 (0.00)	0 (0.00)	
Sore throat at days 14	No, n (%)	21 (100.00)	14 (100.00)	N/A
	Yes, n (%)	0 (0.00)	0 (0.00)	
Sore throat at days 30	No, n (%)	11 (100.00)	11 (100.00)	N/A
	Yes, n (%)	0 (0.00)	0 (0.00)	
Diarrhea at baseline period	No, n (%)	25 (96.15)	14 (77.78)	.142*
	Yes, n (%)	1 (3.85)	4 (22.22)	
Diarrhea at day 1	No, n (%)	25 (100.00)	16 (88.89)	.169*
	Yes, n (%)	0 (0.00)	2 (11.11)	
Diarrhea at days 3	No, n (%)	24 (96.00)	16 (88.89)	.562*
	Yes, n (%)	1 (4.00)	2 (11.11)	
Diarrhea at days 5	No, n (%)	23 (92.00)	16 (88.89)	1.000*
	Yes, n (%)	2 (8.00)	2 (11.11)	
Diarrhea at days 7	No, n (%)	24 (100.00)	13 (86.67)	.142#
	Yes, n (%)	0 (0.00)	2 (13.33)	
Diarrhea at days 14	No, n (%)	21 (100.00)	14 (100.00)	N/A
	Yes, n (%)	0 (0.00)	0 (0.00)	
Diarrhea at days 30	No, n (%)	11 (100.00)	10 (90.91)	N/A
	Yes, n (%)	0 (0.00)	1 (9.09)	
Chest tightness at baseline period	No, n (%)	20 (76.92)	9 (50.00)	.064
	Yes, n (%)	6 (23.08)	9 (50.00)	
Chest tightness at day 1	No, n (%)	21 (84.00)	13 (72.22)	.455#
	Yes, n (%)	4 (16.00)	5 (27.78)	
Chest tightness at days 3	No, n (%)	21(84.00)	13 (72.22)	.456#
	Yes, n (%)	4 (16.00)	5 (27.78)	
Chest tightness at days 5	No, n (%)	21 (84.00)	15 (83.33)	1.000#
	Yes, n (%)	4 (16.00)	3 (16.67)	
Chest tightness at days 7	No, n (%)	21 (87.50)	11 (73.33)	.396#
	Yes, n (%)	3 (12.50)	4 (26.67)	
Chest tightness at days 14	No, n (%)	16 (76.19)	13 (92.86)	.366#
	Yes, n (%)	5 (23.81)	1 (7.14)	
Chest tightness at days 30	No, n (%)	9 (81.82)	11 (100.00)	.476#
	Yes, n (%)	2 (18.18)	0 (0.00)	

*Note*.

* Represents significant difference in experimental group and control group (*P* < .05); “n” represents number; “N/A” represents not applicable; “#” represents Fisher's exact test to obtain the *P* value, and others use χ² test without label.

### 3.2 Analysis of the use of concomitant medications

As per the principles of treatment for severe and critically ill COVID‐19 patients, combination drugs were allowed to treat the patients as effectively as possible. Concomitant medications were mainly used for symptomatic treatment, antiviral treatment, antibacterial treatment, hormone therapy, intestinal microbial state regulation, extracorporeal blood purification, traditional Chinese medicinal treatment, and basic disease treatment, among others. As shown in Table 3, there were no statistical differences in the types of combined medications between the two groups (*P* > .05). However, there were intragroup differences with regard to concomitant treatment using extracorporeal blood purification both in the experimental group (*P* < .001) and in the control group (*P* = .015). Specifically, concomitant treatment with an extracorporeal blood purification system was employed more often for critically ill patients than for severe patients in both the experimental and the control groups.

**TABLE 3:** Reasons for concomitant medication use for the 44 COVID‐19 patients in the experimental group and the control group

	Experimental group (N = 26)			Control group (N = 18)			
Category	Severe (N = 16)	Critically ill (N = 10)	*P* ^a^	Severe (N = 10)	Critically ill (N = 8)	*P* ^b^	*P* ^c^
Symptomatic treatment, n (%)	14 (87.50)	10 (100.00)	.508	10 (100.00)	8 (100.00)	N/A	.505
Antiviral therapy, n (%)	15 (93.75)	8 (80.00)	.538	10 (100.00)	8 (100.00)	N/A	.258
Antibacterial treatment, n (%)	9 (56.25)	6 (60.00)	1.000	8 (80.00)	6 (75.00)	1.000	.167
Hormone, n (%)	7 (43.75)	7 (70.00)	.248	7 (70.00)	7 (87.50)	.588	.105
Gut microflora modulator, n (%)	1 (6.25)	0 (0.00)	1.000	3 (30.00)	1 (12.50)	.588	.142
Extracorporeal blood purification system, n (%)	1 (6.25)	9 (90.00)	<.001*	2 (20.00)	7 (87.50)	.015*	.447
Traditional Chinese medicine treatment, n (%)	9 (56.25)	6 (60.00)	1.000	6 (60.00)	7 (87.50)	.314	.325
Basic disease medication, n (%)	2 (12.50)	3 (30.00)	.340	2 (20.00)	5 (62.50)	.145	.183

*Note*.

* Represents significant difference (*P* < .05); *P* ^a^ value is the experimental group of the difference between severe and critically ill patients in each type of combination medication in the experimental group; *P* ^b^ is the difference test between severe and critically ill patients in each type of combination medication in the control group; *P* ^c^ is the test group in each type of combination medication test the difference with the control group (do not distinguish the severity of the disease); “n” represents number; “N/A” represents not applicable.

### 3.3 Assessment of the efficacy of MSC infusion after 1‐month follow‐up

**FIGURE 2:**
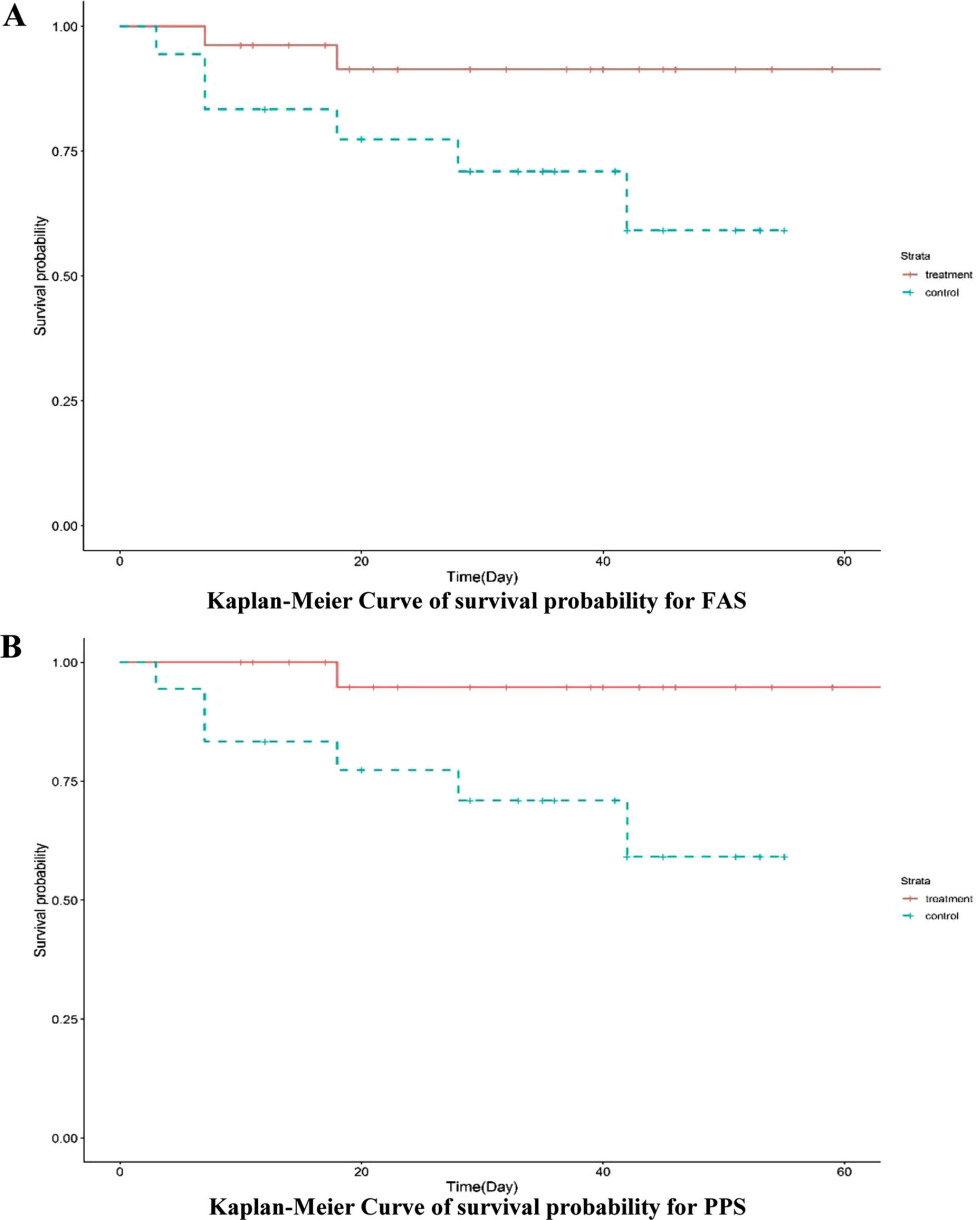
Kaplan–Meier Curve of survival probability for the FAS (A) and the PPS (B) analysis. The experimental group showed better survival than the control group in both analysis sets, suggesting that MSC transplantation improved the survival rate of COVID‐19 patients

The primary endpoint for this study was the survival rate of severe and critical COVID‐19 patients with or without MSC infusion. Treatment efficacy was assessed throughout the study period both in the FAS and the PPS. As shown in Table 4, the survival rate for the experimental group was 92.31% (24/26) for the FAS, while the survival rate for the control group was 66.67% (12/18). This difference in survival rate was statistically significant (*P* = .048). Similar results were obtained when the PPS was analyzed separately. The survival curves for the FAS and the PPS are presented in Figure 2A, B, respectively. Our results suggest that MSC infusion exerts a positive therapeutic effect on the survival rate of severe and critically ill COVID‐19 patients.

**TABLE 4:** Survival status of 44 COVID‐19 patients in the experimental group and the control group

	FAS			PPS		
Survival status	Experimental group (N = 26)	Control group (N = 18)	*P*	Experimental group (N = 24)	Control group (N = 18)	*P*
Survival, n (%)	24 (92.31)	12 (66.67)	.048*	23 (95.83)	12 (66.67)	.031*
Death, n (%)	2 (7.69)	6 (33.33)		1 (4.17)	6 (33.33)	

*Note*.

* Represents significant difference (*P* < .05); FAS, full analysis set; PPS, per protocol set; “n” represents number; *P* value represents the survival rate of COVID‐19 patients in the experimental group and the control group based on FAS and PPS analysis in the entire period.

For severe patients, a Cox proportional hazards model of the FAS showed that the hazards ratio (HR) for the experimental group relative to the control group was .10 (95% CI, 0.00‐1284.41), which was not statistically significant (*P* = .437; Supporting information Table S3). Using gender and age as covariates, the adjusted HR (95% CI) was .00 (0.00–∞), which was also not statistically significant (*P* = .970). For critically ill patients, the HR (95% CI) for the experimental group relative to the control group was .34 (.06‐1.88; *P* = .218), and the adjusted HR (95% CI) with gender and age as covariates was .11 (.01‐.89), which was statistically significant (*P* = .039). Similar results were obtained when the PPS was analyzed (Supporting information Table S4): the adjusted HR (95% CI) with gender and age as covariates was .12 (0.00–.95), which was statistically significant (*P* = .047). Thus, our results suggest that MSC transplantation increases survival more for critically ill patients than for severe patients when adjusting for gender and age.

**FIGURE 3:**
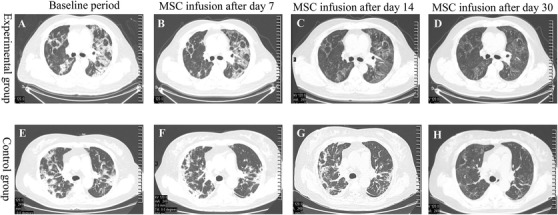
Representative chest CT images at various points during 1‐month follow‐up after MSC transplantation. Images shown are from baseline (A), day 7 (B), day 14 (C), and day 30 (D) following MSC transplantation in the experimental group as well as baseline (E), day 7 (F), day 14 (G), and day 30 (H) of the observation period for the control group. There was a statistically significant difference between the groups in the improvement rate of chest imaging results in the first month after MSC infusion. Representative CT images clearly show improved results at days 7, 14, and 30 for both groups

The secondary endpoints for this study mainly included negative viral test results, time taken to recover from all symptoms, length of hospital stay, number of days in the ICU, occurrence of shock, incidence of multiple organ failure, change in chest CT results, and respiratory support status. Detailed results are presented in Table 5. The average time taken to recover from viral infection, as indicated by a negative viral test, was 15.75 ± 13.71 and 18.31 ± 9.86 days for the experimental and the control groups, respectively. Statistical analysis showed that these differences were not significant (*P* = .251). The average time to improvement for the experimental group and the control group was 3.00 ± 3.05 and 8.80 ± 10.77 days, respectively, meaning that the average time taken to improve for the experimental group was 5.8 days shorter than that for the control group, and this difference was statistically significant (*P* = .049), indicating that MSC infusion was able to shorten the time required for treatment. There was no significant difference in either the length of hospital stay or in the number of days in the ICU (both *P* > .05), and there was also no significant difference in the occurrence of shock or multiple organ failure between the groups (both *P* > .05). Further, there was no statistical difference in the use of respiratory support between the two groups (Table 6). One month after MSC infusion, 20 patients in the experimental group and 12 patients in the control group underwent chest CT by three respiratory physicians who were blinded to the treatment group; 17 (85.00%) patients in the experimental group had improved, while 3 (15.00%) patients showed no significant change. In contrast to the experimental group, six (50.00%) patients in the control group had improved, while the other six (50.00%) showed no significant change. Figure 3 shows representative CT scans documenting lung improvement at various time points for both the control and the experimental group. The difference in the improvement of chest imaging results in the first month after MSC infusion was significant. Representative CT images of both groups at post‐treatment days 7, 14, and 30 are shown in Figure 3. Together, these results suggest that the relative improvement rate was higher for the experimental group during the 1 month after MSC infusion than it was for the control group.

**TABLE 5:** Secondary endpoints used as efficacy indicators for the 44 COVID‐19 patients in the experimental group and the control group

Index	Experimental group	Control group	*P*
Virus negative time (Mean ± SD, days)	15.75 ± 13.71	18.31 ± 9.86	.251
Time to improve (Mean ± SD, days)	3.00 ± 3.05	8.80 ± 10.77	.049
The number of days in hospital (Mean ± SD, days)	30.65 ± 16.16	34.94 ± 18.00	.413
Stay in ICU (Mean ± SD, days)	24.00 ± 12.67	22.17 ± 20.66	.465
Shock			1.000
Yes, n (%)	4 (15.38)	3 (16.67)	
No, n (%)	22 (84.62)	15 (83.33)	
Multiple organ dysfunction syndrome (MODS)			.128
Yes, n (%)	3 (11.54)	6 (33.33)	
No, n (%)	23 (88.46)	12 (66.67)	
Rate of chest imaging changes			.049*
n for check (n for not check)	20 (6)	12 (6)	
Improvement, n (%)	17 (85.00)	6 (50.00)	
No change, n (%)	3 (15.00)	6 (50.00)	
Exacerbation, n (%)	0 (0.00)	0 (0.00)	

*Note*. “n” represents number.

* Represents significant difference (*P* < .05).

**TABLE 6:** Respiratory function support indicators of 44 COVID‐19 patients in experimental group and control group

Interview	Indicator	Condition	Experimental group (N = 26)	Control group (N = 18)	*P*
Screening period	Noninvasive ventilation therapy	Yes, n (%)	1 (3.85)	2 (11.11)	.558
		No, n (%)	25 (96.15)	16 (88.89)	
	Intubation assisted ventilation	Yes, n (%)	4 (15.38)	2 (11.11)	1.000#
		No, n (%)	22 (84.62)	16 (88.89)	
	Extracorporeal membrane oxygenation (ECMO)	Yes, n (%)	0 (0.00)	0 (0.00)	N/A
		No, n (%)	26 (100.00)	18 (100.00)	
MSCs infusion at day 1	Noninvasive ventilation therapy	Yes, n (%)	1 (3.85)	4 (22.22)	.142#
		No, n (%)	25 (96.15)	14 (77.78)	
	Intubation assisted ventilation	Yes, n (%)	3 (11.54)	3 (16.67)	.676#
		No, n (%)	23 (88.46)	15 (83.33)	
	Extracorporeal membrane oxygenation (ECMO)	Yes, n (%)	0 (0.00)	0 (0.00)	N/A
		No, n (%)	26 (100.00)	18 (100.00)	
MSCs infusion at days 3	Noninvasive ventilation therapy	Yes, n (%)	1 (3.85)	2 (11.11)	.558#
		No, n (%)	25 (96.15)	16 (88.89)	
	Intubation assisted ventilation	Yes, n (%)	3 (11.54)	3 (16.67)	.676#
		No, n (%)	23 (88.46)	15 (83.33)	
	Extracorporeal membrane oxygenation (ECMO)	Yes, n (%)	0 (0.00)	1 (5.56)	.409#
		No, n (%)	26 (100.00)	17 (94.44)	
MSCs infusion at days 5	Noninvasive ventilation therapy	Yes, n (%)	1 (3.85)	2 (11.11)	.558#
		No, n (%)	25 (96.15)	16 (88.89)	
	Intubation assisted ventilation	Yes, n (%)	4 (15.38)	3 (16.67)	1.000#
		No, n (%)	22 (84.62)	15 (83.33)	
	Extracorporeal membrane oxygenation (ECMO)	Yes, n (%)	0 (0.00)	1 (5.56)	.409#
		No, n (%)	26 (100.00)	17 (94.44)	
MSCs infusion after days 7± 1	Noninvasive ventilation therapy	Yes, n (%)	0 (0.00)	2 (13.33)	.135#
		No, n (%)	25 (100.00)	13 (86.67)	
	Intubation assisted ventilation	Yes, n (%)	4 (16.00)	4 (26.67)	.444#
		No, n (%)	21 (84.00)	11 (73.33)	
	Extracorporeal membrane oxygenation (ECMO)	Yes, n (%)	0 (0.00)	1 (6.67)	.375#
		No, n (%)	25 (100.00)	14 (93.33)	
MSCs infusion after days 14± 3	Noninvasive ventilation therapy	Yes, n (%)	0 (0.00)	1 (6.67)	.375#
		No, n (%)	25 (100.00)	14 (93.33)	
	Intubation assisted ventilation	Yes, n (%)	4 (16.00)	4 (26.67)	.444#
		No, n (%)	21 (84.00)	11 (73.33)	
	Extracorporeal membrane oxygenation (ECMO)	Yes, n (%)	1 (4.00)	0 (0.00)	1.000#
		No, n (%)	24 (96.00)	15 (100.00)	
MSCs infusion after days 30 ± 3	Noninvasive ventilation therapy	Yes, n (%)	0 (0.00)	1 (7.69)	.351#
		No, n (%)	24 (100.00)	12 (92.31)	
	Intubation assisted ventilation	Yes, n (%)	2 (8.33)	4 (30.77)	.157#
		No, n (%)	22 (91.67)	9 (69.23)	
	Extracorporeal membrane oxygenation (ECMO)	Yes, n (%)	0 (0.00)	0 (0.00)	N/A
		No, n (%)	24 (100.00)	13 (100.00)	

*Note*. “n” represents number; “#” represents Fisher's exact test to obtain the *P* value, and others use *χ*² test without label; “N/A” represents not applicable.

Additionally, inflammatory indices (including CRP and IL‐6) and oxygenation indices (FiO_2_, SpO_2_, SaO_2_, and PaO_2_) were analyzed before and after MSC infusion (Table 7), and there were no significant differences in CRP (*P* = .486), IL‐6 (*P* = .375), FiO_2_ (*P* = .174), and SaO_2_ (*P* = .068). Interestingly, SpO_2_ was significantly improved following MSC infusion, from 94.72 ± 3.4% before treatment to 96.04 ± 5.93% after treatment (*P* < .001). Moreover, PaO_2_ was significantly improved following MSC transplantation, from 78.89 ± 25.86 mmHg before treatment to 95.62 ± 39.49 mmHg after treatment (*P* = .015).

**TABLE 7:** Inflammatory indices (including CRP and IL‐6) and oxygenation indices (FiO_2_, SpO_2_, SaO_2_, and PaO_2_) before and after MSCs infusion

Index (unit)		Before MSCs treatment	After MSCs treatment	*P*
CRP (mg/L)	N	65	229	.486
	Mean ± SD	42.27 ± 57.70	36.62 ± 52.66	
	Median (Min‐Max)	13.84 (.41‐200)	11.18 (.05‐200)	
IL‐6 (pg/mL)	N	40	182	.375
	Mean ± SD	86.31 ± 250.79	352.28 ± 2953.53	
	Median (Min‐Max)	10.72 (.46‐1436.82)	13.16 (1.50‐39225.30)	
FiO2 (%)	N	9	14	.174
	Mean ± SD	58.78 ± 24.28	49.57 ± 23.05	
	Median (Min‐Max)	49.00 (33.00‐102.00)	40.00 (29.00‐100.00)	
SpO_2_ (%)	N	41	68	<.001*
	Mean ± SD	94.72 ± 3.40	96.04 ± 5.93	
	Median (Min‐Max)	95.40 (86.00‐99.20)	98.00 (60.00‐100.00)	
SaO_2_ (%)	N	10	19	.068
	Mean ± SD	93.50 ± 3.89	90.95 ± 17.70	
	Median (Min‐Max)	94.00 (84.00‐99.00)	97.00 (30.00‐100.00)	
PaO_2_ (mmHg)	N	44	56	.015*
	Mean ± SD	78.89 ± 25.86	95.62 ± 39.49	
	Median (Min‐Max)	70.50 (45.00‐146.00)	91.25 (24.00‐208.00)	

*Note*.

* Represents significant difference (*P* < .05) by the Wilcoxon rank sum test. “N” represents the number of valid observation data. CRP, C‐reactive protein; FiO2, fraction of inspiration O2; IL‐6, interleukin 6; Min, minimum; Max, maximum; PaO2, partial pressure of oxygen; SaO2, oxygen saturation; .SpO2, peripheral oxygen saturation.

### 3.4 Presence of AEs following treatment

The severity of each AE related to COVID‐19 infection was graded from 1 to 5 for both the experimental group and the control group, and a summary of all AEs experienced by the 44 COVID‐19 patients included in the current study are presented in Supporting information Table S5. According to the statistical analysis, 76.92% (20/26) of the patients experienced a total of 56 AEs in the experimental group, including 40 AEs of grade 1, 6 AEs of grade 2, 3 AEs of grade 3, 4 AEs of grade 4, and 3 AEs of grade 5. In contrast, 100.00% (18/18) of the patients experienced a total of 59 AEs in the control group, including 39 AEs of grade 1, 5 AEs of grade 2, 3 AEs of grade 3, 7 AEs of grade 4, and 5 AEs of grade 5 (Supporting information Table S5). During the study period, no patients in either the experimental group or the control group showed AEs that necessitated withdrawal from the study. A detailed analysis of the AEs observed in the experimental group and the control group is presented in Table 8. As shown in Table 8, 10 of the 56 AEs observed in the experimental group were grade 3 or higher, while 15 of the 59 AEs observed in the control group were grade 3 or higher. Except for a difference in the incidence of high blood pressure between the two groups, there were no significant differences (*P* > .05) between the groups for any AEs related to the measured clinical indices including the blood test results, liver function markers, blood lipid levels, renal function, myocardial enzymes, electrolyte disturbances, and clinical symptoms. High blood pressure was observed in two (3.57%) patients in the experimental group and six (10.18%) patients in the control group (*P* = .048). In summary, the frequency of each AE was statistically similar between the two groups, except for the AE related to high blood pressure, which was more common in the control group. Further, the experimental group showed a lower incidence of AEs (76.92%) than the experimental group (100.00%), but the difference was not statistically significant. Together, these results suggest that the MSC infusion protocol used in this study showed good safety outcomes.

**TABLE 8:** Frequency of each adverse event (AE) for the 44 COVID‐19 patients in the experimental group and the control group

	Experimental group (N = 26)		Control group (N = 18)		
Condition of AEs	Total (%)	≥Grade 3	Total (%)	≥Grade 3	*P*
**Blood routine**					
Increased CRP	1 (1.79)	0	0 (0)	0	1.000
Leukopenia	3 (5.35)	0	0 (0)	0	.258
Thrombocytopenia	1 (1.79)	0	0 (0)	0	1.000
Thrombocytosis	0 (0)	0	1 (1.69)	0	.409
**Liver function**					
Abnormal liver function	4 (7.14)	1	3 (5.09)	1	1.000
Elevated bilirubin	0 (0)	0	1 (1.69)	0	.409
Hypoproteinemia	2 (3.57)	0	1 (1.69)	0	1.000
Increased alkaline phosphatase	0 (0)	0	1 (1.69)	0	.409
**Blood lipids**					
Elevated triglycerides	3 (5.35)	0	4 (6.79)	0	.419
Elevated cholesterol	4 (7.14)	0	0 (0)	0	.133
Hyperlipidemia	2 (3.57)	0	1 (1.69)	0	1.000
**Renal function**					
Increased creatinine	1 (1.79)	0	2 (3.39)	1	.409
Elevated uric acid	0 (0)	0	1 (1.69)	0	.409
**Myocardial enzymes**					
Increased creatine kinase	1 (1.79)	0	0 (0)	0	1.000
Elevated lactate dehydrogenase	1 (1.79)	0	0 (0)	0	1.000
**Coagulation**					
Abnormal blood clotting function	2 (3.57)	0	5 (8.48)	1	1.000
Prothrombin time prolonged	0 (0)	0	1 (1.69)	0	.409
Increased fibrinogen	1 (1.79)	0	0 (0)	0	1.000
Elevated blood sugar	2 (3.57)	0	0 (0)	0	.505
**Electrolyte disturbance**					
Hypocalcemia	1 (1.79)	0	0 (0)	0	1.000
Hypokalemia	2 (3.57)	0	2 (3.39)	0	1.000
Low chlorine	0 (0)	0	2 (3.39)	0	.162
Low sodium	0 (0)	0	2 (3.39)	0	.162
Elevated blood sodium	0 (0)	0	1 (1.69)	0	.409
Hyperkalemia	2 (3.57)	0	1 (1.69)	0	1.000
**Clinical symptoms**					
Fever	1 (1.79)	0	3 (5.09)	0	.289
Diarrhea	1 (1.79)	0	1 (1.69)	0	1.000
Expiratory dyspnea	2 (3.57)	1	0 (0)	0	1.000
Respiratory failure	1 (1.79)	1	0 (0)	0	.505
Cough	3 (5.35)	0	0 (0)	0	.258
Anemia	5 (8.92)	0	7 (11.88)	0	.273
Heart failure	0 (0)	0	1 (1.69)	1	.409
Chest tightness	1 (1.79)	0	1 (1.69)	0	1.000
High blood pressure	2 (3.57)	0	6 (10.18)	0	.048*
ARDS	1 (1.79)	1	2 (3.39)	2	.558
Shock	3 (5.35)	3	3 (5.09)	3	.676
Gastrointestinal bleeding	1 (1.79)	1	1 (1.69)	1	1.000
Multifunctional organ failure	2 (3.57)	2	5 (8.48)	5	.103
Total times	56 (100)	10	59 (100)	15	

*Note*. Fisher's exact test to obtain the *P* value. ARDS, acute respiratory distress syndrome.

* Represents significant difference (*P* < .05).

## 4 DISCUSSION

The initial symptoms of COVID‐19 are often fever, cough, sputum, and shortness of breath. These in turn can lead to dyspnea, ARDS, lung injury, shock, and eventual multiple organ failure.^68^, ^69^ An autopsy study by Xu et al reported that a deceased COVID‐19 patient showed a large amount of sputum, presumably causing severe ARDS, as well as a large number of inflammatory factors in the lung tissue.^13^ This suggests that effective treatment of ARDS and prevention of multiple organ failure is a key strategy in preventing mortality in COVID‐19 patients. Severe ARDS causes breathing difficulties, and it has been suggested that resolving breathing difficulties in COVID‐19 patients in a timely manner may make it is possible to inhibit COVID‐19 progression. Interestingly, we found that patients treated with MSCs experienced immediate and dramatic relief from breathing difficulties associated with COVID‐19 on day 1 (*P* = .016), day 3 (*P* = .040), and day 5 (*P* = .031) compared with the control group. Further, our results show that both SpO_2_ and PaO_2_, indices associated with oxygenation levels, were significantly improved after MSC infusion. These results further support the use of MSC infusion as a method to combat ARDS and expiratory dyspnea, particularly for critically ill COVID‐19 patients.

Multiple complications of the COVID‐19 epidemic make it difficult to fully treat patients with this disease. Many interventions in China and around the world have proven effective to reduce the epidemic and prevent the virus from continuing to spread.^5^, ^70^, ^71^, ^72^, ^73^, ^74^ Although effective social distancing can mitigate the virus's persistence, treating COVID‐19 is also an important strategy toward ending the current pandemic.

X‐ray and chest CT imaging results of COVID‐19 patients in the ICU have been shown to reveal the presence of pneumonia, known as novel coronavirus pneumonia. These imaging results reveal bilateral, multilobular involvement as well as subsegmental consolidation.^75^ In the present study, we observed a statistically significant difference in the rate of improvement of chest CT results in the first month after MSC infusion. A total of 85.00% of patients with MSC treatment showed improved chest CT results, compared with only 50.00% of patients in the control group. These results provide further evidence for the efficacy of MSC infusion.

Currently, an effective vaccine would be the best way to combat SARS‐CoV‐2 infection, and many groups have presented preliminary basic and clinical data related to vaccine development.^24^, ^25^, ^76^, ^77^, ^78^ However, assessing the safety and efficacy of any vaccine will take a relatively long period of time. Apart from targeted vaccine development, other therapeutic strategies are being developed in the race against time to end the global pandemic. Remdesivir, an inhibitor of the viral RNA‐dependent, targets nascent viral RNA chains, resulting in premature termination of the viral life cycle.^23^, ^79^ Beigel et al reported that remdesivir can significantly shorten the recovery time of COVID‐19 patients with lower respiratory tract infections in a double‐blind, randomized controlled clinical study.^80^ Corticosteroids are important immunomodulators for the clinical treatment of SARS.^81^ Studies have shown that compared with patients with mild to moderate disease, patients with severe SARS‐CoV‐2 have higher levels of proinflammatory cytokines in serum samples.^82^ A meta‐analysis indicated that the mortality rate was reduced in severe COVID‐19 patients treated with corticosteroids.^83^ More recently, the recovery collaborative group reported that dexamethasone (a type of corticosteroid) significantly decreased 28‐day mortality in patients hospitalized with COVID‐19.^22^ Corticosteroids have therefore become a potential treatment for COVID‐19 patients. Compared with remdesivir or corticosteroids, MSC infusion has the potential to significantly reduce dyspnea in a relatively short period of time, as MSCs act by targeting secretory factors and MSC‐released extracellular vesicles deliver microRNA, mRNA, or DNA.^84^, ^85^ The main methods currently employed to treat COVID‐19 patients (especially severe and critically ill patients) include the following: (1) convalescent plasma therapy; (2) antiviral drug therapy; (3) traditional Chinese and western medicine; (4) MSC‐based therapy; and (5) immune‐mediated therapy.^86^, ^87^, ^88^, ^89^, ^90^, ^91^ Studies on these methods have accelerated the screening of effective drugs, explored new treatment methods, and attempted to prevent mild cases from progressing in severity.

The current clinical study reports that MSC infusion enhanced the survival rate for severe or critically ill COVID‐19 patients in both the FAS (92.31% survival in the experimental group vs 66.67% in the control group; *P* = .048) and the PPS (95.83% survival in the experimental group vs. 66.67% in the control group; *P* = .031). Our results agree with a previous report detailing the potential for MSC infusion to treat critically ill COVID‐19 patients by alleviating acute respiratory dysfunction and pulmonary fibrosis.^91^ There are some other sources of MSCs used in clinical studies for treating COVID‐19, and these are included in a systematic review and meta‐analysis.^92^, ^93^, ^94^ Leng et al^53^ used UC‐MSCs to treat seven COVID‐19 patients, and had three control patients who were infected with COVID‐19; there was 100% survival in the experimental group versus 66.67% survival in the control group. Shu et al^95^ investigated 12 severe COVID‐19 patients using UC‐MSCs as the experimental group, and 29 severe COVID‐19 patients using a placebo as the control group. Their results showed a 100% 28‐day survival rate in the experimental group versus 89.66% survival in the control group. More recently, Meng et al^57^ performed a study in 18 hospitalized patients with moderate to severe COVID‐19 pulmonary disease, 9 of whom were treated with UC‐MSC infusions and with 9 control patients. All patients survived both in the experimental group and in the control group, but the degree of severity of COVID‐19 (moderate to severe) might have affected the outcome. Leng et al,^53^ Shu et al,^95^ and our study enrolled more severe or critically ill COVID‐19 patients. Recently, Sánchez‐Guijo et al^96^ investigated 13 severe COVID‐19 patients using an AD‐MSCs infusion for a clinical study that was nonrandomized and without a control; the results showed 84.62% survival using an AD‐MSCs infusion. Therefore, MSC transplantation from different sources appears to be a candidate method to improve outcomes for critical cases. Coagulopathy and thromboprophylaxis are very common after MSC infusion.^15^ Current clinical data indicate that MSCs from human menstrual blood does not clot in patients, which is very favorable for intravenous infusions. One possible reason could be that MSCs express a low level of procoagulant tissue factor TF/CD142, which needs to be systematically investigated and verified in future preclinical studies. Although different sources of MSCs have been investigated for effectiveness in treating COVID‐19, more optimized treatment strategies are critical to evaluate and control blood compatibility, optimize cell transfusion, and monitor the real‐time dynamics of cells in the body to develop safer and more effective MSC treatments.^43^ Several concomitant treatments have been shown to exert a synergistic role with MSC transplantation. Of note, Peng et al^97^ reported that intravenous infusion of convalescent plasma as a treatment for severe COVID‐19 may have synergistic characteristics with MSC transplantation in inhibiting cytokine storms, promoting lung injury repair, and recovery of pulmonary function. Although further study is required to establish safety and efficacy, the current body of evidence suggests that MSC transplantation might be an effective treatment for severe and critical COVID‐19.

In the current study, it was observed that many clinical symptoms were ameliorated in the 1‐month period following MSC transplantation with combined therapy. No cases of pulmonary embolism were observed in the patients who underwent MSC infusion, although this side‐effect is considered to be the main concern regarding MSC safety. Rather, our results indicate that MSC therapy is a safe and effective therapeutic strategy to rescue severe and critical lung problems induced by SARS‐CoV‐2. Thus, in the present study, preliminary clinical data were provided with regard to the short‐term safety (1‐month follow‐up) and therapeutic effect of MSC transplantation to treat severe and critically ill patients.

The current study does not provide long‐term evidence related to MSC‐induced AEs. In a previous report from our group, MSCs were used to treat 17 H7N9 patients and four of those patients were followed up for 5 years without observing any AEs.^55^ In the current study of severe and critical COVID‐19 patients, almost no significant differences in the occurrence of AEs in the short term were observed between the control group and the group receiving MSC transplantation. Hence, this study found that MSC infusion is associated with good safety outcomes. In addition, although the potential application value of MSC treatment in COVID‐19 is obvious, the innate and adaptive immune compatibility test of MSC is incorporated into the current cell detection system, and the establishment of strict monitoring standards for biosafety and effectiveness with regard to the coagulopathy and thromboprophylaxis is crucial.^15^ Researchers should also pay strict attention to obtaining a suitable source, the product quality, monitoring of the various physiological and biochemical indicators of patients throughout the process, and strict prevention of unnecessary safety hazards.^98^

Recently, Leng et al^53^ published a clinical study on using MSCs to treat COVID‐19 patients. This study investigated inflammatory and immune functioning as well as adverse effects in seven patients for 14 days post‐MSC transplantation. The authors reported that MSCs appeared to significantly improve the functional outcomes of all seven patients without any observed AEs. However, more clinical data are still needed to identify any short‐term adverse reactions following MSC administration. Zheng et al^99^ recently reported that 12 patients with moderate to severe ARDS did not experience any infusion‐related reactions or serious treatment‐related AEs following MSC transplantation. Additionally, Meng et al^57^ performed a study that included 18 hospitalized patients with moderate to severe COVID‐19 pulmonary disease, nine of whom were treated with UC‐MSC infusions. According to their results, intravenous UC‐MSC infusion was safe and well‐tolerated throughout the 1‐month follow‐up period. Although long‐term follow‐up data regarding the tolerability and safety of MSC transplantation are lacking, MSC transplantation may still be an effective method treatment for COVID‐19, especially for severe and critical cases.

There are several limitations to this exploratory trial. First and foremost, one patient experienced an AE over grade 3 associated with severe abnormal liver function. More data from a larger study are needed to determine whether MSC infusion can lead to an infusion reaction in certain patients, especially as infusion reactions can cause shortness of breath. Since only 26 patients were treated with MSCs in the current study and because we used a multicenter, open, and parallel‐controlled study design, our study may not have detected important AEs associated with MSC treatment. Secondly, we should stress that this clinical trial did not use a standard design owing to the unique nature of the COVID‐19 outbreak and the ethical limitations associated with treating severe COVID‐19 patients. Thirdly, we reported that the experimental group showed greater improvements in CT results than the control group during the 1‐month study period. However, we only observed significant improvements in expiratory dyspnea for the experimental group versus the control group on days 1, 3, and 5, and no differences were observed between the groups at other time points with regard to lung function. We speculate that MSCs exert a short‐term effect to improve expiratory dyspnea, while the long‐term improvements were more due to the actions of concomitant medications. Moreover, we did not observe significant changes in the levels of inflammatory factors following MSC infusion, even though mortality was significantly decreased in the experimental group. Therefore, the mechanism by which MSCs reduce mortality should be investigated in future more comprehensive clinical trials. Moll et al investigated that the differences between fresh and cryopreserved MSCs were limited but significant.^100^ Fresh MSCs are the best choices, however, considering the COVID‐19 outbreak, the lack of sufficient donors for providing menstrual blood in a short time, and the consistent standard of care between the two hospitals situated in different cities, freshly thawed MSCs were used in this study. Finally, the small sample size of the current study limited our ability to obtain enough clinical data to draw strong conclusions.

## 5 CONCLUSIONS

This prospective and systematic study assessed the ability of menstrual blood‐derived MSCs to treat both severe and critically ill COVID‐19 patients. The results of this multicenter, open, and parallel‐controlled clinical study suggest that menstrual blood‐derived MSC transplantation significantly lowers the mortality of severe and critical SARS‐CoV‐2‐induced COVID‐19. Menstrual blood‐derived MSCs may act by alleviating the breathing difficulties caused by COVID‐19 and reducing the symptoms of ARDS or expiratory dyspnea. Although the body of research on MSCs is still in its infancy and lacks important long‐term safety information, MSC‐based therapy may serve in future clinical applications as an alternative method for the treatment of COVID‐19.

## AUTHOR CONTRIBUTIONS

Charlie Xiang, Lanjuan Li, Lingling Tang, and Yingan Jiang conceived and designed this study; Xiaowei Xu, Wanli Jiang, Lijun Chen, and Zhenyu Xu performed the experiments, collected and analyzed the data, and wrote the manuscript. Qiang Zhang, Mengfei Zhu, Peng Ye, Hang Li, Liang Yu, Xiaoyang Zhou, Chenliang Zhou, Xiaobei Chen, Xiaoqin Zheng, Kaijin Xu, Hongliu Cai, Shufa Zheng, Wubian Jiang, Xiaojun Wu, Dong Li, Lu Chen, Qingqing Luo, Yingyan Wang, Jingjing Qu, Yifei Li, and Wendi Zheng collected and analyzed the data. All authors have read and approved this final manuscript.

## AVAILABILITY OF DATA AND MATERIALS

All data analyzed in this study are included in this published article.

## CONFLICT OF INTEREST

The authors declare that they have no competing interests.

## ETHICS APPROVAL AND CONSENT TO PARTICIPATE

This study was submitted and approved by the Ethics Committee of the First Affiliated Hospital, Collage of Medicine, Zhejiang University, Hangzhou, China and the Ethics Committee of Renmin Hospital of Wuhan University (WDRY2020‐K011), Wuhan, China. MSC administration in patients with COVID‐19 was conducted in a multicenter and open‐label clinical trial (ChiCTR2000029606).

## Supporting information

Supporting Information

Supporting Information
