# Supplementary material for: Evaluation of the safety and efficacy of using human menstrual blood‐derived mesenchymal stromal cells in treating severe and critically ill COVID‐19 patients: An exploratory clinical trial
Source: Clin Transl Med. 2021 Jan 27;11(2):e297. doi: 10.1002/ctm2.297 (PMC7839959; doi:10.1002/ctm2.297)
Supplement: Supplementary file 1 — Supporting Information [file CTM2-11-e297-s001.docx]

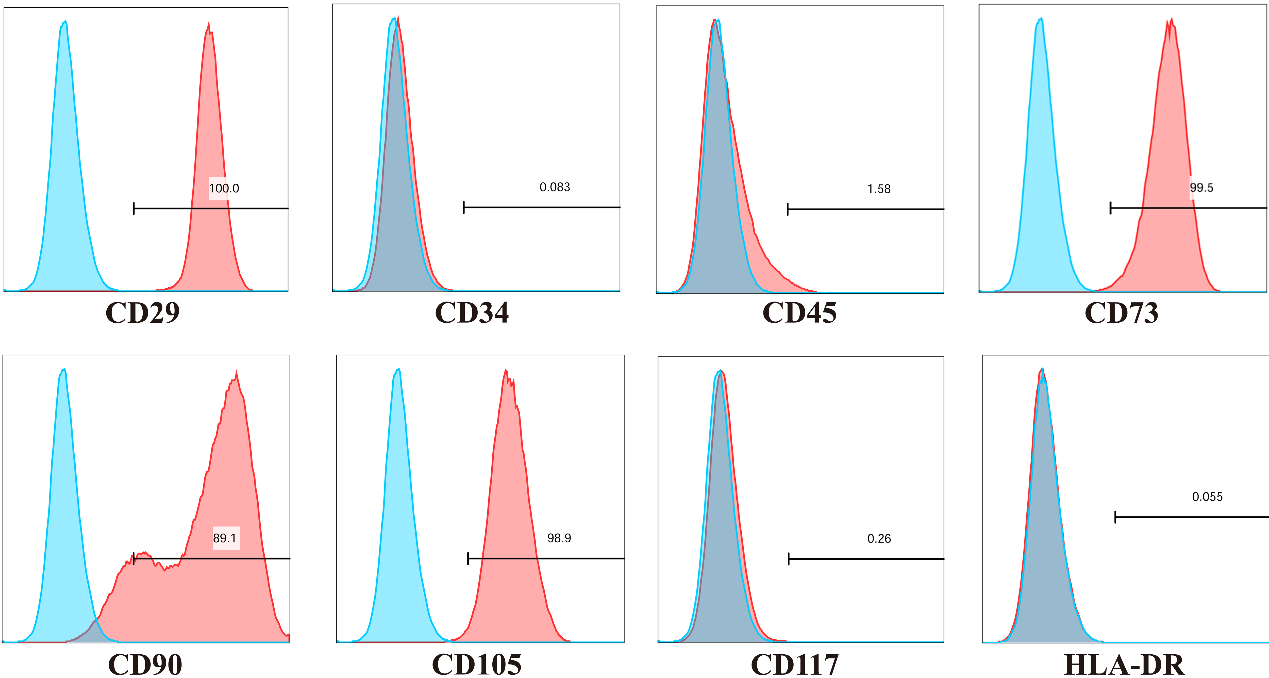


**Figure S1**. Characterization of cell surface markers in MSCs. Representative images clearly show that MSCs strongly expressed CD29, CD73, CD90, and CD105; and MSCs were negative for CD34, CD45, CD117, and human leukocyte antigen-DR (HLA-DR); blue represents isotype control and red represents PE stained samples.

**
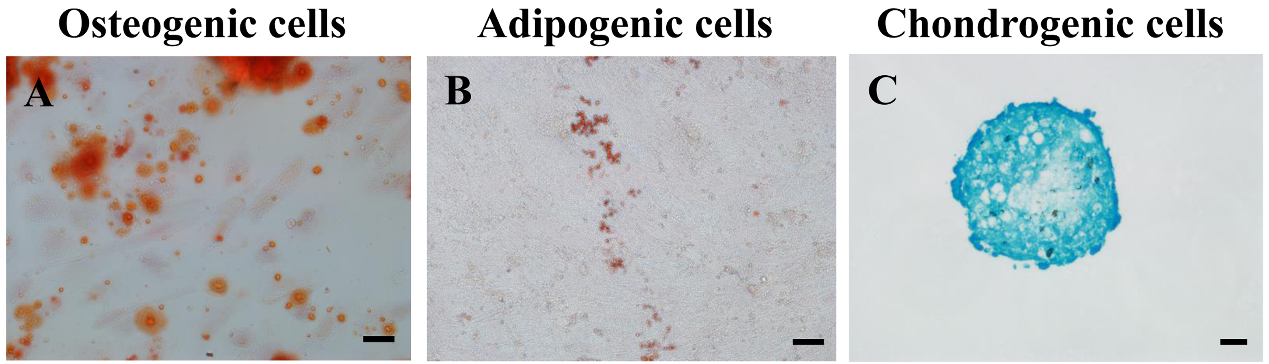
**

**Figure S2**. The representative picture for MSCs induced into osteogenic, adipogenic, and chondrogenic cells. (A) Alizarin red staining of calcium deposition for differentiated osteogenic cells; (B) Oil Red O staining of intracellular neutral lipid vacuoles for differentiated adipogenic cells; (C) Alcian blue staining of sulfated cartilage for differentiated chondrogenic cells. Scale bar represents 100 μm for each lane.

**
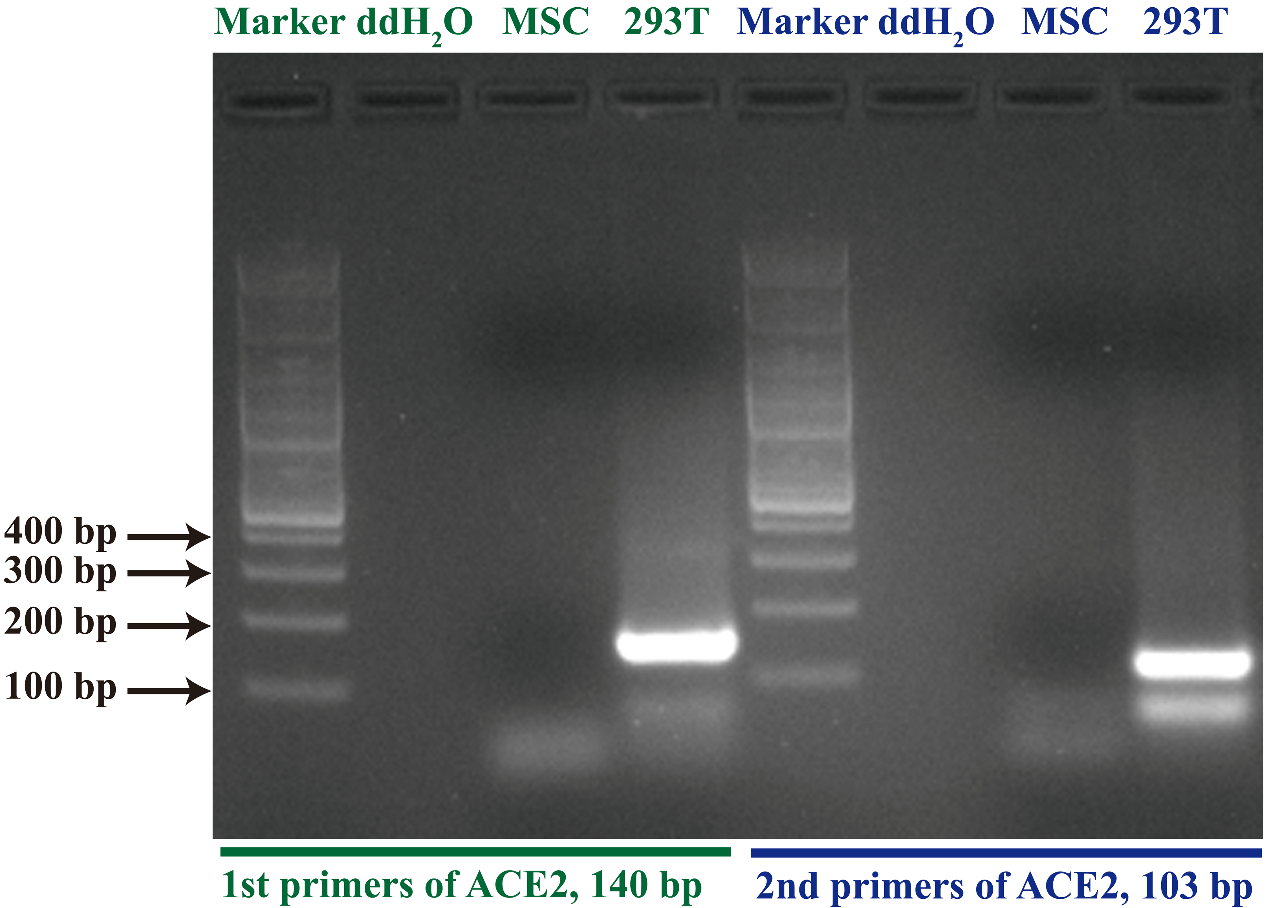
**

**Figure S3**. Characterization of the ACE2 expression level in MSC. A representative electrophoretogram with two pairs of primers shown by PCR analysis. MSC ACE2 expression was negative according to PCR analysis, with ddH_2_O as a negative control and 293T cells as a positive control.
