# Supplementary material for: Evaluation of the safety and efficacy of using human menstrual blood‐derived mesenchymal stromal cells in treating severe and critically ill COVID‐19 patients: An exploratory clinical trial
Source: Clin Transl Med. 2021 Jan 27;11(2):e297. doi: 10.1002/ctm2.297 (PMC7839959; doi:10.1002/ctm2.297)
Supplement: Supplementary file 2 — Supporting Information [file CTM2-11-e297-s002.docx]

**Table S1**. Antibodies used for the flow cytometry in the study.

| **Antibody name** | **Supplier** | **Catalog No.** | **Usage** | **Dosage** |
| --- | --- | --- | --- | --- |
| PE mouse anti-human CD29 | BD Biosciences | 561795 | FACS | 10 μL |
| PE mouse anti-human CD34 | BD Biosciences | 560941 | FACS | 10 μL |
| PE mouse anti-human CD45 | BD Biosciences | 560975 | FACS | 10 μL |
| PE mouse anti-human CD73 | BD Biosciences | 561014 | FACS | 10 μL |
| PE mouse anti-human CD90 | BD Biosciences | 561970 | FACS | 2.5 μL |
| PE mouse anti-human CD105 | BD Biosciences | 560839 | FACS | 2.5 μL |
| PE mouse anti-human CD117 | BD Biosciences | 561682 | FACS | 2.5 μL |
| PE mouse anti-human HLA-DR | BD Biosciences | 560943 | FACS | 10 μL |
| PE mouse IgG1 | BD Biosciences | 555749 | FACS | 10 μL |
| PE mouse IgG2a | BD Biosciences | 555574 | FACS | 10 μL |

**Note:** The dilution of antibody follows the instruction. HLA-DR, human leukocyte antigen-DR isotype; FACS, fluorescence-activated cell sorting.

**Table S2**. Primers used for PCR analysis.

| **Gene name** | **Sequence (5’ to 3’)** | **Fragment size (bp)** | **Tm (℃)** | **Accession No.** |
| --- | --- | --- | --- | --- |
| ACE2-F-1 | CGAAGCCGAAGACCTGTTCTA | 161 | 60 | AB046569 |
| ACE2-R-1 | GGGCAAGTGTGGACTGTTCC |  |  |  |
| ACE2-F-2 | ACAGTCCACACTTGCCCAAAT | 103 | 60 | AB046569 |
| ACE2-R-2 | TGAGAGCACTGAAGACCCATT |  |  |  |

**Note:** F, forward; R, reverse. ACE2, angiotensin-converting enzyme 2.

**Table S3**. Cox proportional hazards model table for factors affecting survival time with FAS analysis.

| **Classification** | **Factors** | **Before adjustment** |  | **After adjustment** **&** |  |
| --- | --- | --- | --- | --- | --- |
|  |  | **HR (95%CI)** | ***P*** | **HR (95%CI)** | **P** |
| Severe (%) | Control group | N/A | N/A | N/A | N/A |
|  | Experimental group | 0.10 (0.00~1284.41) | 0.437 | 0.00 (0.00~∞) | 0.970 |
| Critically ill (%) | Control group | N/A | N/A | N/A | N/A |
|  | Experimental group | 0.34 (0.06~1.88) | 0.218 | 0.11 (0.01~0.89) | 0.039* |

Note: “*” represents significant difference; “N/A” represents not applicable; “&” represents after adjustment by the gender and age.

**Table S4**. Cox proportional hazards model table for factors affecting survival time with PPS analysis.

| **Classification** | **Factors** | **Before adjustment** |  | **After adjustment** **&** |  |
| --- | --- | --- | --- | --- | --- |
|  |  | **HR (95%CI)** | ***P*** | **HR (95%CI)** | **P** |
| Severe (%) | Control group | N/A | N/A | N/A | N/A |
|  | Experimental group | 0.10 (0.00~1284.41) | 0.437 | 0.00 (0.00~∞) | 0.970 |
| Critically ill (%) | Control group | N/A | N/A | N/A | N/A |
|  | Experimental group | 0.21 (0.02~1.85) | 0.158 | 0.12 (0.00~0.95) | 0.047* |

Note: “*” represents significant difference; “N/A” represents not applicable; “&” represents after adjustment by the gender and age.

**Table S5**. The summary of the severity of adverse events (AEs) of 44 COVID-19 patients in experimental group and control group

| **Indicator** | **Experimental group (N=26)** | | | | | | **Control group (N=18)** | | | | | |
| --- | --- | --- | --- | --- | --- | --- | --- | --- | --- | --- | --- | --- |
|  | **Numbers** | **Severity (grade)** | | | | | **Numbers** | **Severity (grade)** | | | | |
|  |  | **1** | **2** | **3** | **4** | **5** |  | **1** | **2** | **3** | **4** | **5** |
| **ARDS** | 1 | 0 | 0 | 0 | 1 | 0 | 2 | 0 | 0 | 0 | 2 | 0 |
| **Increased CRP** | 1 | 1 | 0 | 0 | 0 | 0 | 0 | 0 | 0 | 0 | 0 | 0 |
| **Leukopenia** | 3 | 3 | 0 | 0 | 0 | 0 | 0 | 0 | 0 | 0 | 0 | 0 |
| **Elevated cholesterol** | 4 | 4 | 0 | 0 | 0 | 0 | 0 | 0 | 0 | 0 | 0 | 0 |
| **Elevated bilirubin** | 0 | 0 | 0 | 0 | 0 | 0 | 1 | 0 | 1 | 0 | 0 | 0 |
| **Hypoproteinemia** | 2 | 2 | 0 | 0 | 0 | 0 | 1 | 1 | 0 | 0 | 0 | 0 |
| **Hypocalcemia** | 1 | 1 | 0 | 0 | 0 | 0 | 0 | 0 | 0 | 0 | 0 | 0 |
| **Hypokalemia** | 2 | 2 | 0 | 0 | 0 | 0 | 2 | 2 | 0 | 0 | 0 | 0 |
| **Low chlorine** | 0 | 0 | 0 | 0 | 0 | 0 | 2 | 2 | 0 | 0 | 0 | 0 |
| **Low sodium** | 0 | 0 | 0 | 0 | 0 | 0 | 2 | 2 | 0 | 0 | 0 | 0 |
| **Multiple organ failure** | 2 | 0 | 0 | 0 | 1 | 1 | 5 | 0 | 0 | 0 | 2 | 3 |
| **Fever** | 1 | 1 | 0 | 0 | 0 | 0 | 3 | 3 | 0 | 0 | 0 | 0 |
| **Diarrhea** | 1 | 1 | 0 | 0 | 0 | 0 | 1 | 0 | 1 | 0 | 0 | 0 |
| **Elevated triglycerides** | 3 | 3 | 0 | 0 | 0 | 0 | 4 | 4 | 0 | 0 | 0 | 0 |
| **Abnormal liver function** | 4 | 3 | 0 | 1 | 0 | 0 | 3 | 2 | 0 | 1 | 0 | 0 |
| **Hyperkalemia** | 2 | 1 | 1 | 0 | 0 | 0 | 1 | 0 | 1 | 0 | 0 | 0 |
| **Hyperlipidemia** | 2 | 2 | 0 | 0 | 0 | 0 | 1 | 1 | 0 | 0 | 0 | 0 |
| **Expiratory dyspnea** | 2 | 1 | 0 | 1 | 0 | 0 | 0 | 0 | 0 | 0 | 0 | 0 |
| **Respiratory failure** | 1 | 0 | 0 | 0 | 0 | 1 | 0 | 0 | 0 | 0 | 0 | 0 |
| **Increased creatinine** | 1 | 0 | 1 | 0 | 0 | 0 | 2 | 1 | 0 | 1 | 0 | 0 |
| **Increased creatine kinase** | 1 | 1 | 0 | 0 | 0 | 0 | 0 | 0 | 0 | 0 | 0 | 0 |
| **Increased alkaline phosphatase** | 0 | 0 | 0 | 0 | 0 | 0 | 1 | 1 | 0 | 0 | 0 | 0 |
| **Cough** | 3 | 3 | 0 | 0 | 0 | 0 | 0 | 0 | 0 | 0 | 0 | 0 |
| **Elevated uric acid** | 0 | 0 | 0 | 0 | 0 | 0 | 1 | 1 | 0 | 0 | 0 | 0 |
| **Abnormal blood clotting function** | 2 | 1 | 1 | 0 | 0 | 0 | 5 | 2 | 2 | 0 | 1 | 0 |
| **Prothrombin time prolonged** | 0 | 0 | 0 | 0 | 0 | 0 | 1 | 1 | 0 | 0 | 0 | 0 |
| **Anemia** | 5 | 4 | 1 | 0 | 0 | 0 | 7 | 7 | 0 | 0 | 0 | 0 |
| **Elevated lactate dehydrogenase** | 1 | 0 | 1 | 0 | 0 | 0 | 0 | 0 | 0 | 0 | 0 | 0 |
| **Increased fibrinogen** | 1 | 1 | 0 | 0 | 0 | 0 | 0 | 0 | 0 | 0 | 0 | 0 |
| **Gastrointestinal bleeding** | 1 | 0 | 0 | 1 | 0 | 0 | 1 | 0 | 0 | 1 | 0 | 0 |
| **Heart failure** | 0 | 0 | 0 | 0 | 0 | 0 | 1 | 0 | 0 | 0 | 1 | 0 |
| **Chest tightness** | 1 | 1 | 0 | 0 | 0 | 0 | 1 | 1 | 0 | 0 | 0 | 0 |
| **Shock** | 3 | 0 | 0 | 0 | 2 | 1 | 3 | 0 | 0 | 0 | 1 | 2 |
| **Elevated blood sodium** | 0 | 0 | 0 | 0 | 0 | 0 | 1 | 1 | 0 | 0 | 0 | 0 |
| **Elevated blood sugar** | 2 | 2 | 0 | 0 | 0 | 0 | 0 | 0 | 0 | 0 | 0 | 0 |
| **Thrombocytopenia** | 1 | 0 | 1 | 0 | 0 | 0 | 0 | 0 | 0 | 0 | 0 | 0 |
| **Thrombocytosis** | 0 | 0 | 0 | 0 | 0 | 0 | 1 | 1 | 0 | 0 | 0 | 0 |
| **High blood pressure** | 2 | 2 | 0 | 0 | 0 | 0 | 6 | 6 | 0 | 0 | 0 | 0 |
| **Total** | 56 | 40 | 6 | 3 | 4 | 3 | 59 | 39 | 5 | 3 | 7 | 5 |
